# Refractory massive chylothorax following robot-assisted laparoscopic splenectomy with pericardial devascularization treated with trans-jugular intrahepatic portosystemic shunt: a case report

**DOI:** 10.3389/fmed.2024.1420157

**Published:** 2024-09-04

**Authors:** Xiang Deng, Jun Xia

**Affiliations:** Department of Abdominal Surgery, Guiqian International Hospital, Guiyang, China

**Keywords:** LSPD, portal hypertension, gastric fundus varices, massive chylothorax, tips

## Abstract

The development of a chylothorax after robot-assisted laparoscopic splenectomy combined with pericardial devascularization (LSPD) is rare. The robot-assisted procedure is similar to the standard LSPD, but surgeons must remain vigilant about potential chylothorax caused by recurrence of portal hypertension in patients with cirrhosis, an event that leads to variceal bleeding in the gastric fundus or a massive chylothorax caused by a thoracic duct fistula. We report a rare case of massive chylothorax after robot-assisted LSPD and review the literature to help elucidate the mechanisms of portal hypertension after LSPD, reduce surgical complications, and improve long-term patient outcomes. After LSPD, portal pressure monitoring, coagulation function testing, and portal vein CT imaging help in excluding portal vein thromboses and ensuring appropriate anticoagulation to reduce the development of thoracic duct fistulas. If portal hypertension recurs after surgery and a high-output chylothorax develops, conservative treatment becomes ineffective. Treatment with an active trans-jugular intrahepatic portosystemic shunt (TIPS) is recommended to lower the portal pressure.

## 1 Introduction

Liver cirrhosis is a common chronic wasting disease. The clinical symptoms of an acute decompensation include mainly ascites, splenomegaly, and esophageal varices. In addition, thrombocytopenia caused by hypersplenism and esophageal variceal bleeding caused by portal hypertension seriously affect the quality of life of patients with liver cirrhosis and even threaten their lives (1–3). Laparoscopic splenectomy combined with pericardial devascularization (LSPD) is often performed in patients with liver cirrhosis accompanied by severe portal hypertension. This procedure can effectively reduce the portal pressure and the risk of gastrointestinal bleeding without interfering with the blood supply to the liver, reducing the impact on the patients’ postoperative liver function. In addition, the operation can relieve hypersplenism, restore platelet levels, and improve the coagulation function. LSPD is a minimally invasive procedure with favorable therapeutic effects (4–6). Although platelet levels increase after LSPD, secondary portal vein thrombosis may occur in patients without adequate coagulation function, further increasing the portal pressure and increasing abdominal lymphatic reflux (7), which may lead to the development of thoracic duct fistulas. In this article, we report the development of a thoracic duct fistula after robot-assisted LSPD, and we detail its treatment methods.

## 2 Case report

**Figure 1:**
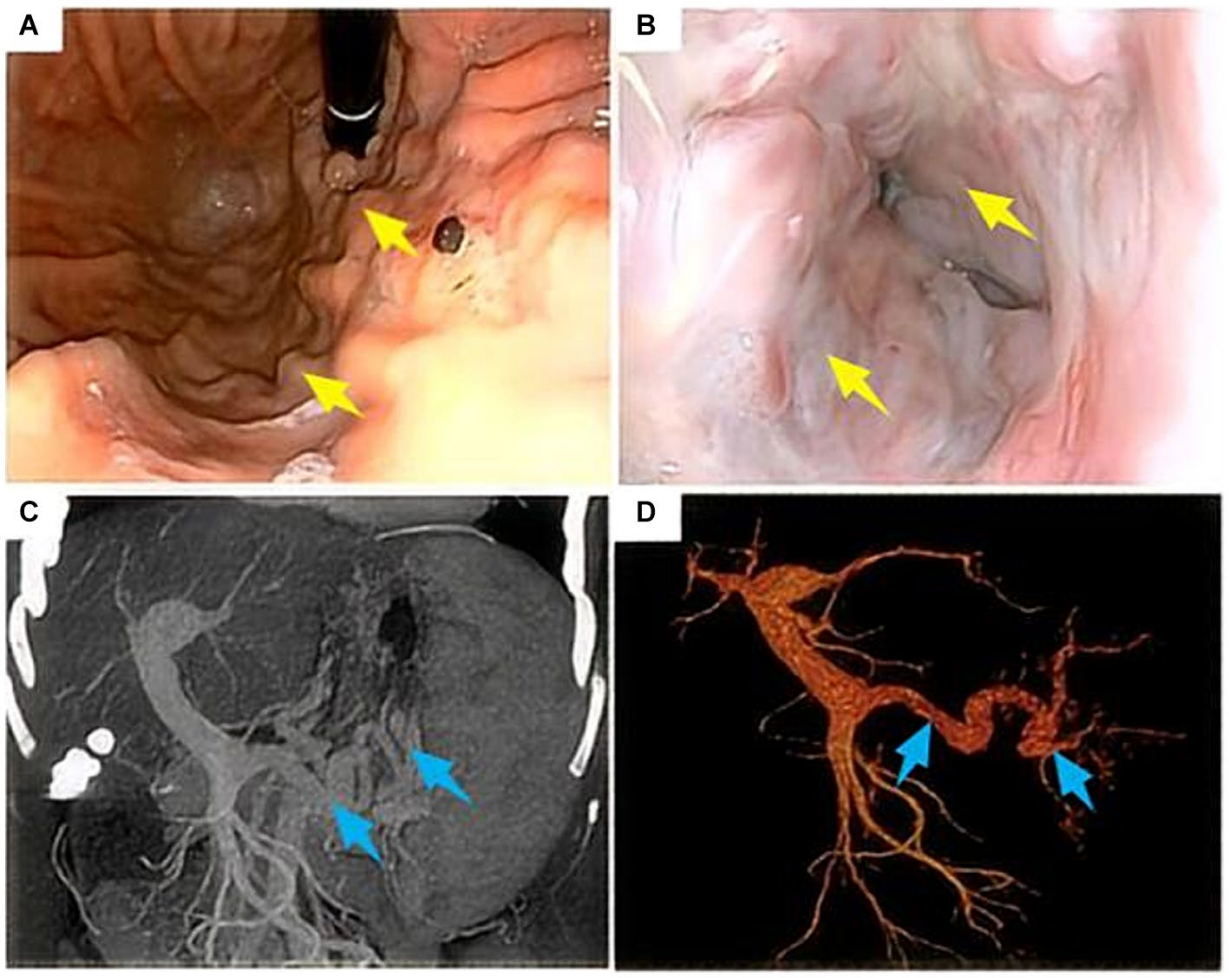
Gastroduodenoscopy and computed tomography scan of the upper abdomen in a 47-year-old female patient who had been vomiting blood repeatedly for 6 years before admission. **(A,B)** Gastroduodenoscopy suggests severe varices of the esophageal and fundal veins (yellow arrows); **(C,D)** CT scan of the upper abdomen suggests cirrhosis, splenomegaly, and portal hypertension (widening of the main portal vein and varicose splenic veins) (blue arrows).

A 47-year-old woman presented for a physical examination with the chief complaint of “Hepatitis C for 10 years and recurrent hematemesis for 6 years.” She had been treated with medication in a local hospital without regular antiviral therapy, and had recurrent symptoms of anorexia and fatigue, without abdominal pain or distension, palpitations, or chest tightness. Her physical examination showed a temperature of 36.5°C, blood pressure at 132/54 mmHg, pulse at 70 beats/min, respiration at 20 beats/min, normal consciousness, a slightly poor mental state, an anemic appearance, no abnormalities on auscultation of heart and lungs, no jaundice, and a soft abdomen without tenderness or rebound tenderness. The spleen was palpable 5 cm below the costal margin, and she had no edema in either leg. She had had hepatitis C for 10 years without other special medical conditions. There were no similar diseases in the family and no family genetic diseases. From 2015 to 2021, she underwent endoscopic ligation and blood transfusion therapy 6 times due to variceal bleeding in the gastric fundus, but the condition still relapsed. On July 23, 2021, she was admitted to the Hepatobiliary Surgery department for treatment based on her medical history and clinical symptoms. According to the 2023 “Endoscopic Diagnosis and Treatment of Esophageal and Gastric Variceal Bleeding: European Society of Gastrointestinal Endoscopy Guidelines” (8), she was diagnosed as having “cirrhosis with gastric fundus varices and hepatitis C cirrhosis” and was admitted for treatment. Upon admission, she underwent relevant examinations, and a gastrointestinal endoscopy revealed severe esophagogastric varices and portal hypertension gastropathy (Figures 1A,B). Plain and contrast-enhanced scans of the upper abdominal vein, hepatic artery, and inferior vena cava revealed the presence of liver cirrhosis (maximum oblique diameter of the right lobe, 10.25 cm; thickness and length of the left lobe, 4.24 cm and 6.12 cm, respectively; thickness of the right lobe, 9.13 cm), splenomegaly (longitudinal diameter and thickness of the spleen, 14.22 cm and 7.98 cm, respectively), portal hypertension (main trunk of the portal vein, 1.58 cm), and splenic varices (Figures 1C,D). Blood routine examination (red blood cell count, 3.66 * 1012/L; hemoglobin, 82 g/L; platelet count, 64 * 109/L), coagulation function (prothrombin time, 13.0 s; international normalized ratio 1.14; activated partial thromboplastin time, 25.7 s; thrombin time, 14.9 s; and thrombinogen quantitative dimer, 0.46 μg/mL) and liver and kidney function tests, and electrolyte and tumor markers were all normal.

**Figure 2:**
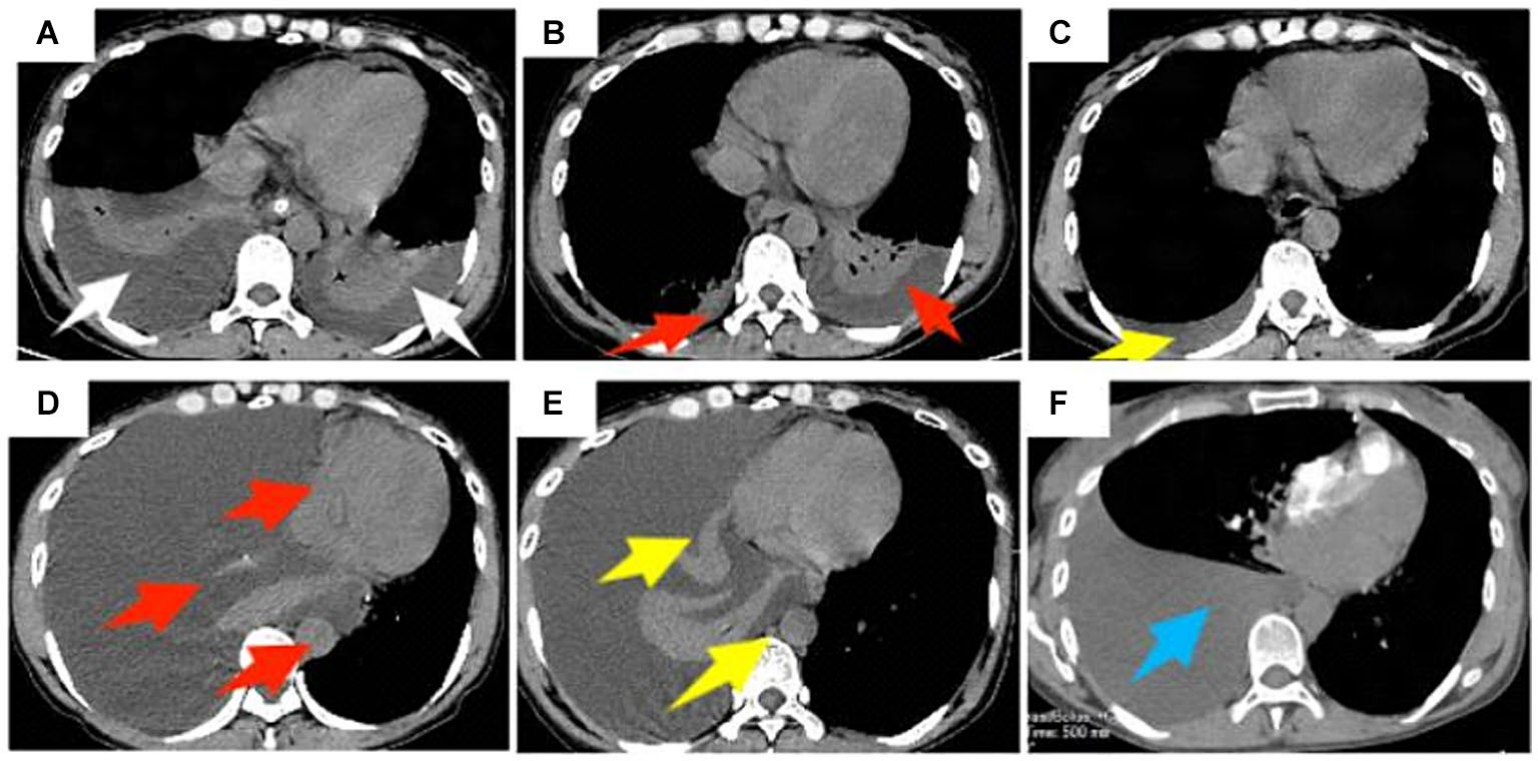
A 47-year-old female patient underwent obot-assisted laparoscopic splenectomy combined with pericardial devascularization (LSPD). **(A)** Chest CT 4 days after LSPD showed bilateral pleural effusions and most of the lower lobes of both lungs were atelectatic (white arrows); **(B)** percutaneous percutaneous drainage of the right pleural effusion was performed, and a follow-up CT 8 days after the operation showed a decrease in the bilateral pleural effusions (red arrows); **(C)** Chest CT 47 days after the operation showed a small amount of effusion in the right pleural cavity (yellow arrow); **(D)** The mediastinal window of the chest CT on day 116 postoperatively showed a large right pleural effusion, complete atelectasis of the right lung, and left shift of the trachea, heart, and mediastinum (red arrow); **(E)** the patient underwent percutaneous right pleural effusion puncture and drainage, and chest CT on day 129 postoperatively still showed a large right pleural effusion (yellow arrow); **(F)** chest CT on day 137 postoperatively showed a large right pleural effusion but less than before (blue arrow).

Combining the patient’s medical history and clinical auxiliary examination results, we attributed the patient’s repeated hematemesis to a portal hypertension caused by liver cirrhosis, resulting in upper gastrointestinal bleeding. The patient’s main request is for treatment of upper gastrointestinal bleeding. After completing the preoperative examinations to rule out surgical contraindications, the patient underwent robot-assisted LSPD under general anesthesia on July 30, 2021 (operation duration, 5 h and 15 min; intraoperative bleeding, 800 mL including 600 mL of splenic blood; intraoperative infusion, 800 mL of suspended red blood cells, 600 mL of plasma, and 400 mL of platelets). After the operation, the patient was given intensive respiratory, circulatory, and coagulation function monitoring, gastrointestinal decompression, infection prevention, liver protection, inhibition of gastric acid secretion, and nebulization/inhalation therapy. On the fourth postoperative day, the patient suddenly felt chest tightness and underwent a chest CT, which showed bilateral pleural effusions, atelectasis in most of the lower lobes of both lungs, and a small amount of pericardial effusion (Figure 2A). The patient underwent ultrasound-guided percutaneous right pleural effusion puncture, and the puncture fluid was a clear, pale yellow liquid with a negative chyle test (−). After the puncture, the daily drainage volume was approximately 200 mL, and the patient continued to receive the previous treatment, in addition to antiviral therapy. Eight days after the operation, CT reexamination images showed that the bilateral pleural effusions had decreased (Figure 2B). Continued symptomatic supportive treatment resulted in a stable condition and improved mental state on the 11th post-operative day. The chest drainage tube was removed and the patient was discharged.

After discharge, the patient recovered smoothly. Later, 47 days after the LSPD, chest CT images showed only a small amount of right-sided pleural effusion (Figure 2C). At 116 days after the LSPD, the patient experienced severe coughing and felt chest tightness and shortness of breath. Chest CT reexamination showed a large amount of right-sided pleural effusion, complete atelectasis of the right lung, and left displacement of the trachea, heart, and mediastinum (Figure 2D). The patient was admitted to the hepatobiliary surgery department again for an ultrasound-guided thoracentesis. The puncture fluid was milky white and turbid, and the chylous test result was positive (+). The daily drainage volume after puncture was approximately 1,500 mL. At this time, the patient’s liver function was still normal [total bilirubin (TBIL), 8.6 μmol/L; alanine aminotransferase (ALT), 18 U/L; aspartate transaminase (AST), 18 U/L; albumin (ALB), 42.3 g/L]. However, 129 days after the LSPD, the chest CT images still showed a large right-sided pleural effusion (Figure 2E). The patient developed hypoalbuminemia (ALB, 31.4 g/L) and moderate peritoneal effusion, which was considered related to the chylothorax. Liver protection, hypoalbuminemia correction, moderate diuresis, nutritional support, and other treatments were continued. The daily drainage volume of chest drainage fluid decreased to approximately 1,000 mL, and the amount of peritoneal effusion decreased gradually. On postoperative day 137, a chest CT showed a large pleural effusion on the right side that was smaller than the previous one (Figure 2F). Conservative treatment was continued with fasting, nutritional support, continuous chest drainage, and intrathoracic injection of pingyangmycin (16 mg of pingyangmycin +10 mg of dexamethasone, chest injection, 5–7 days/time, continuous treatment for 3 times) taking into consideration the massive chylothorax of the patient. However, the effect was poor, and the chest drainage fluid increased to approximately 4,000 mL/day. The drainage fluid was a milky white turbid liquid, and the chyle test was positive (+).

**Figure 3:**
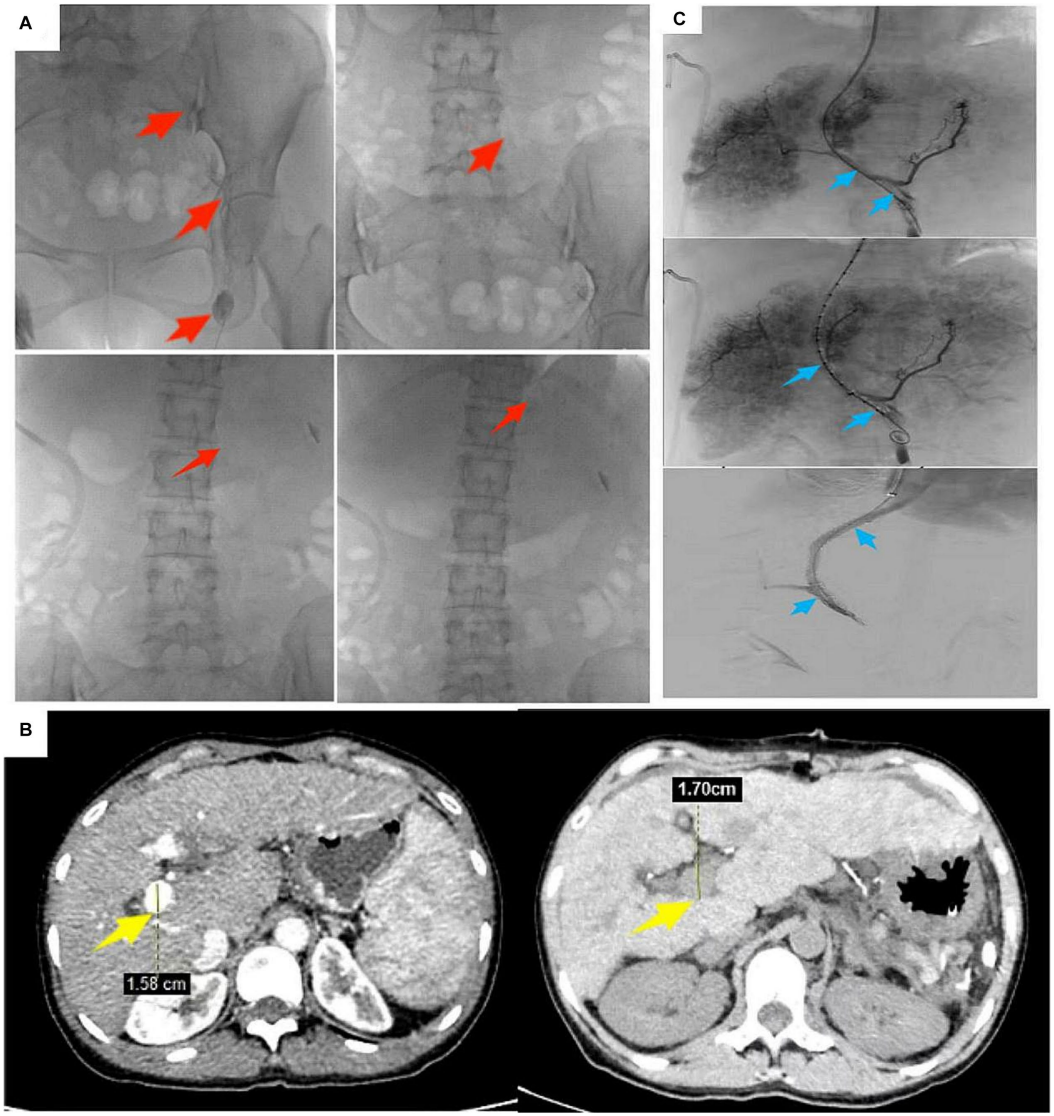
A 47-year-old female patient underwent robot-assisted LSPD. **(A)** 165 days after LSPD, ultrasound-guided lymphangiography through the inguinal region showed no abnormalities in the shape of the pelvic and lumbar lymphatic vessels, and no visualization of the thoracic lymphatic vessels (red arrows); **(B)** Compared with the pre-LSPD, the portal vein width before TIPS increased from 1.58 cm to 1.7 cm (yellow arrow); **(C)** During TIPS treatment, thrombosis in the portal vein was found. The patient underwent TIPS treatment under general anesthesia 169 days after LSPD (blue arrow).

**Figure 4:**
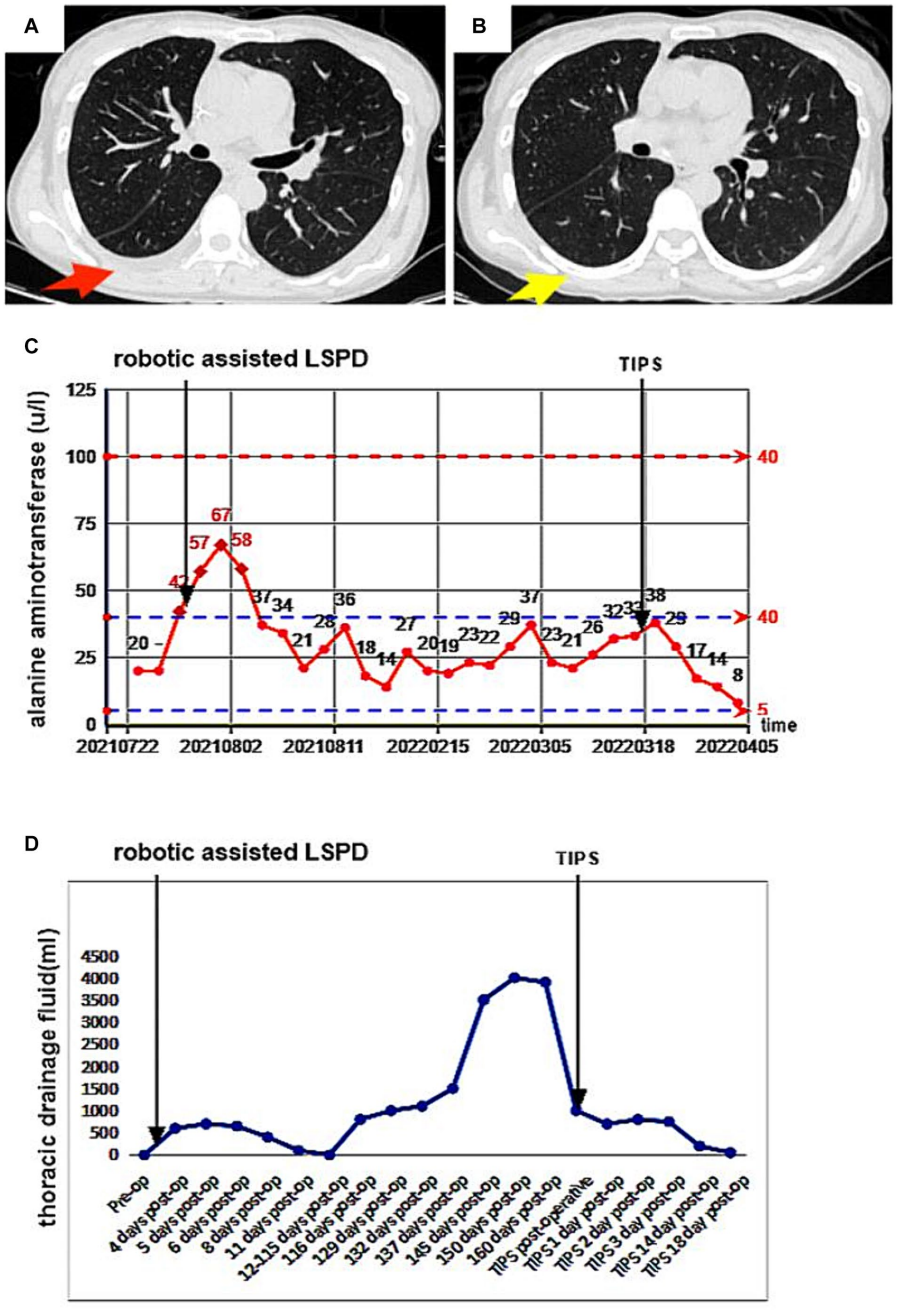
A 47-year-old female patient underwent robotic assisted LSPD, TIPS treatment was performed 169 days after LSPD surgery. **(A)** On the 4th day after TIPS surgery, a chest CT scan (chest window) showed a small amount of pleural effusion on the right side, which was significantly improved compared to before (red arrow); **(B)** 14 days after TIPS surgery, chest CT showed no obvious effusion in the right chest cavity and good lung expansion (yellow arrow); **(C,D)** The patient’s serum alanine aminotransferase (ALT) level and chest drainage fluid decreased significantly after TIPS.

We thought the large right-sided chylothorax was probably associated with the liver cirrhosis, portal hypertension, increased lymphatic fluid production, and lymphatic vessel ruptures. Therefore, we once again excluded surgical contraindications, and the patient underwent ultrasound-guided left lymph node imaging through the inguinal region to identify the location of the chylous fistula. The imaging results showed normal morphology and lymphatic vessels course in the pelvic and lumbar segments, and we found no significant fistulas in the thoracic segment (Figure 3A). Compared with the preoperative LSPD, the portal vein width had increased from 1.58 cm to 1.7 cm (Figure 3B). After a multidisciplinary consultation meeting (thoracic surgery, cardiovascular medicine, interventional therapy room, digestive medicine, and hepatobiliary surgery), we agreed that a trans-jugular intrahepatic portosystemic shunt (TIPS) would be used to reduce the portal pressure and alleviate the lymphatic reflux and the chylothorax. We communicated with the patient’s family and obtained a signed surgical consent form, the patient underwent TIPS under general anesthesia on day 169 after the initial LSPD (Figure 3C). During the procedure, the portal pressure decreased from 40 cm of water column before surgery to 30 cm of water column after the surgery. In addition, we found irregular filling defects in the main portal vein and left and right branches of the portal vein that appeared to be thrombosis lesions (Figure 3C). On days 1, 2, and 3 after the TIPS operation, the chest drainage tube fluid volumes were 700 mL, 800 mL, and 750 mL, respectively. After therapy for liver protection, electrolyte imbalance correction, anti-infection, and other symptomatic treatments, the chest CT images showed improvement in the right-sided pleural effusion compared with the pre-operative findings (Figure 4A). On day 14 after the TIPS, chest CT images showed a small pleural effusion on the right side and local thickening and adhesion of the bilateral pleuras (Figure 4B). The patient’s chest drainage tube fluid decreased to 50 mL 18 days after the TIPS operation, and the chest drainage tube was removed. The patient was discharged 20 days after the TIPS operation and has been followed up for 2 years without recurrence of gastric variceal bleeding or chylothorax. The patient signed a written informed consent for the publication of the data in this case report.

## 3 Discussion

The clinical manifestations of patients with cirrhosis and portal hypertension are mainly splenomegaly, ascites, hematemesis, and black stools. Life-threatening gastrointestinal bleeding may occur in severe cases. Therefore, to ensure the safety of patients, active prevention of complications is necessary during the treatment of the disease (9, 10). Progress in the treatments for patients with cirrhosis and portal hypertension have led to an increase in the number of LSPD procedures performed. LSPD provides advantages like rapid recovery and minimal invasion, preserving the main trunk of the coronary vein and the azygos vein to the maximum extent, and disconnecting the branch vessels leading to the gastric fundus, lower esophagus, and posterior wall of the stomach. The procedure can reduce the risk of postoperative portal hypertension and eliminate esophagogastric varices, a result important for reducing the risk of gastrointestinal bleeding (4, 11, 12).

LSPD is a relatively well-established procedure favored widely by surgeons for its ability to block abnormal collateral circulation vessels, while preserving the normal autologous shunt vessels. Careful dissection is required during the surgical operation to avoid damaging the pancreatic body and tail during the resection of the spleen pedicle. When blocking collateral circulation vessels, maintaining a clear surgical field and protecting the gastric coronary vessels is important. After the operation, patients’ vital signs need to be closely monitored to promptly detect and treat complications. LSPD is a safe and effective operation that improves the liver’s blood supply, promotes its regeneration, improves its function, and delays the progression of cirrhosis. Moreover, LSPD is an ideal treatment option for patients with portal hypertension due to its hemodynamic effects. The patient in this case presented portal hypertension, ascites, splenomegaly, and esophageal and gastric varices due to hepatitis C cirrhosis. The thrombocytopenia-induced hypersplenism and portal hypertension-induced esophageal and gastric varices had long negatively affected the patient’s quality of life, prompting the administration of the LSPD treatment.

The potential postoperative complications after LSPD include portal venous system thrombosis, gastric emptying dysfunction, bleeding, and abdominal infections. Fortunately, the mechanisms underlying these complications are well understood and effective prevention, diagnosis, and treatment strategies against them exist (13–15). However, we found no reports on treatments for massive chylothorax after LSPD. To the best of our knowledge, this is the first report of a diagnosis and treatment plan for a patient with multiple chylous fistulas after robot-assisted LSPD surgery. The conservative treatment for the chylous fistulas (including intrathoracic injection of pingyangmycin and placement of thoracic drainage tubes) was unsuccessful because the patient’s persistent chylothorax was associated with cirrhosis and portal hypertension, leading to the production of large amounts of lymphatic fluid. Therefore, we decided to administer TIPS treatment to cure the chylothorax by reducing the portal pressure and the lymphatic fluid reflux.

Chylothorax due to trauma, tuberculosis, tumors, or lymphatic vessel injuries is a common occurrence in the clinical practice (16–18). In our patient, the uncommonly massive chylothorax developed after abdominal surgery. The thoracic duct remained intact during the operation, and we found no visible lesions. This patient’s idiopathic massive chylothorax after LSPD may have been caused by the following two conditions: (1) Leakage or rupture of the thoracic duct due to the increased lymphatic fluid reflux from the liver and gastrointestinal sources. This, in turn, occurred as a result of the increased portal vein pressure due to liver cirrhosis and portal vein thrombosis (7, 19). (2) Rupture of the thoracic duct due to the increased pleural cavity and internal chest pressure caused by severe coughing, which also compresses the thoracic duct.

Clinically, many thoracic chylous fistulas can be cured with conservative treatments such as a high-protein low-fat diet and gastrointestinal decompression (20). Fasting directly inhibits the intestinal absorption of chylomicrons at their source, while gastrointestinal decompression effectively reduces the intestinal motility. This motility reduction reduces chylomicron absorption by the intestine, effectively reducing the production of chylomicron fluid and promoting the healing of thoracic duct ruptures. Medium-chain triglycerides in nutritional fluids are more soluble in water and body fluids than long-chain triglycerides, and they do not need to pass through the intestinal lymphatic vessels for distribution. Instead medium-chain triglycerides are directly transported into the liver through the portal vein (21), facilitating local healing of the thoracic duct fistula. In our patient, a large amount of chylothorax remained after early fasting and infusion of albumin and other symptomatic treatments. We selected the subsequent treatment approach after careful consideration due to the large size of the lymphatic fistula and the continued production of large amounts of lymphatic fluid.

The surgical indications for patients with chylous fistula after surgery have not been standardized. Singh et al. (22) suggested that surgical intervention should be performed as soon as possible when metabolic disorders and other complications occur 2 weeks after the initiation of a conservative treatment for chylous fistulas, or when the chylous fluid exceeds 1,000 mL after 1 week of treatment. Lymphatic ligation is a common surgical approach. Preoperative lymphangiography or radionuclide lymphography can be used to locate the chylous fistula. Lymphangiography has dual roles in the diagnosis and treatment of chylous fistulas. On the one hand, when the iodinated oil contrast agent reaches the damaged part of the lymphatic vessel, it can produce an inflammatory response promoting granulation tissue proliferation, which is beneficial for lymphatic closure (23). On the other hand, when the lymphatic vessel is found to have a large rupture during the imaging process, lymphatic embolization can be performed after precise localization of the lesion, with a treatment efficiency reaching 89% (24). The minimally invasive nature and effectiveness of lymphangiography and embolization can help avoid the need for reoperation and reduces the rate of perioperative complications. After pinpointing the damaged lymphatic vessel during the operation, the vessel can be repaired or ligated. The wound can be sprayed with medical protein glue and filled with a gelatin sponge, but the operation is difficult and finding the ruptured lymphatic vessel may be hard (25). In this case, ultrasound-guided lymph node puncture and imaging were performed to accurately locate the lymphatic fistula orifice. During the procedure, we found normal morphology and course of the lymphatic vessels in the pelvic and lumbar segments, without visible thoracic lymphatic vessels. We thought it possible that our patient presented congenital anatomical variations in the lymphatic vessels or that the contrast medium was too viscous to allow for visualization of an obvious fistula orifice.

The lymphatic fistula in our patient occurred after a LSPD operation. The patient had underlying hepatitis C, cirrhosis, and portal hypertension, and the portal vein width widened following the operation. TIPS intraoperative angiography confirmed that the patient had portal vein thrombosis, which further increased the portal vein pressure. This may have caused a sharp increase in the return of chylous fluid to the chest lymph nodes. The patient’s daily chylous fluid drainage volume reached 4,000 mL, and conservative medical fluid drainage measures failed making surgical intervention necessary (22). TIPS may be an effective approach for patients with complex and refractory chylothorax after other surgical procedures (16, 26). TIPS can reduce portal vein pressure and decrease lymphatic fluid reflux, both beneficial outcomes that promote the closure of lymphatic vessels (27, 28). In our patient, the portal vein pressure was effectively reduced after the TIPS, and we administered comprehensive measures such as fasting, liver protection, electrolyte imbalance correction, anti-infection, and intravenous nutritional support. Clinical studies have shown that serum ALT levels directly reflect the severity of liver cell injury with a high sensitivity (29, 30). In our patient, the serum ALT levels and the chest drainage tube fluid volume decreased significantly after the TIPS treatment, and her liver function and thoracic duct fistula improved significantly (Figures 4C,D). The portal hypertension in this patient was related to both cirrhosis and postoperative portal vein thrombosis. Therefore, during LSPD procedures, when the conditions allow it, monitoring the portal vein pressure (and performing a portosystemic shunt as necessary) is important to reduce the occurrence of gastrointestinal re-bleeding and lymphatic fistulas. In addition, monitoring platelet levels and portal vein blood flow after splenectomy, as well as implementing effective anti-platelet aggregation therapy, is particularly important. Increases in platelet counts and a decrease in blood flow velocity in the proximal splenic vein after splenectomy may lead to portal vein thrombosis, further increasing portal vein pressure.

To sum up, postoperative thoracic chylous fistulas are one of the rare complications of LSPD, having a low incidence rate. These fistulas are mainly due to increased pressure in the thoracic duct after the operation. Postoperative chylous fistulas in the abdomen can manifest as water electrolyte imbalances, malnutrition, or immune deficiencies, seriously affecting patients’ prognoses. Puncture and chyle fluid tests are the most commonly used methods for diagnosing chyle fistulas clinically; imaging examination can also help to diagnose chyle fistulas after abdominal surgery. Most patients can be cured with dietary management, catheterization and drainage, and prescription medication. However, for patients with liver cirrhosis in whom conservative treatment is insufficient, a timely lymphatic angiography should be performed to visualize the lymphatic fistula and perform an embolization or a TIPS treatment to reduce the lymphatic reflux, promote fistula healing, and improve the quality of life.

## 4 Conclusion

By reviewing this case and studying relevant literature, we refined the following details: (1) Patients with cirrhosis and portal hypertension undergoing LSPD, who develop massive thoracic duct fistulas after surgery, may benefit from blocking lymphangiography therapy if conservative treatment is ineffective. Timely TIPS treatment can reduce lymphatic flow and promote fistula healing in patients with undeveloped lymphatic vessels or poor blocking effects. (2) Patients with splenomegaly, cirrhosis, and portal hypertension who require LSPD should have their portal vein pressure monitored during the operation and the pressure values before and after devascularization should be compared. If the pressure after the operation is significantly higher than normal, preventive TIPS or portosystemic shunt can reduce surgical complications and provide early benefits for patients prone to develop massive chylous fistulas after surgical operations. (3) Regular follow-ups are needed after LSPD to monitor platelet levels, coagulation function, and examine the portal vein by CT to prevent thromboses caused by high platelet counts and slow blood flow in the splenic vein stump, which can lead to portal vein system thrombosis and further portal vein pressure increments. If the at any point the results reveal an abnormal coagulation function, anticoagulant and antiplatelet therapy should be initiated in a timely manner to reduce the risk of portal hypertension thromboses causing a chylous fistula. (4) For hepatitis patients with abnormal liver function tests, such as elevated bilirubin and transaminase levels, regular and long-term antiviral therapy should be emphasized to prevent the transition from hepatitis to cirrhosis. (5) In our patient, her lymphatic fistula was probably caused by the severe coughing she developed after surgery, which led to increased intrathoracic pressure. Therefore, respiratory management should be strengthened for patients after LSPD to avoid coughing and straining to defecate, thereby reducing the risk of chylous fistula. This report expands the understanding of massive thoracic chylous fistula after LSPD and provides options for the treatment of other idiopathic chylous fistulas. The report also serves to share our clinical experience in the diagnosis and treatment of chylous fistulas; however, large-scale studies are needed to provide more effective treatment options for massive thoracic chylous fistula after LSPD.

## Data Availability

The original contributions presented in the study are included in the article/supplementary material, further inquiries can be directed to the corresponding author.
